# Spatial modulation of nanopattern dimensions by combining interference lithography and grayscale-patterned secondary exposure

**DOI:** 10.1038/s41377-022-00774-z

**Published:** 2022-04-08

**Authors:** Zhuofei Gan, Hongtao Feng, Liyang Chen, Siyi Min, Chuwei Liang, Menghong Xu, Zijie Jiang, Zhao Sun, Chuying Sun, Dehu Cui, Wen-Di Li

**Affiliations:** 1 Department of Mechanical Engineering, University of Hong Kong, Hong Kong, China; 2 School of Microelectronics, Southern University of Science and Technology, Shenzhen, China

**Keywords:** Lithography, Sub-wavelength optics, Polymers

## Abstract

Functional nanostructures are exploited for a variety of cutting-edge fields including plasmonics, metasurfaces, and biosensors, just to name a few. Some applications require nanostructures with uniform feature sizes while others rely on spatially varying morphologies. However, fine manipulation of the feature size over a large area remains a substantial challenge because mainstream approaches to precise nanopatterning are based on low-throughput pixel-by-pixel processing, such as those utilizing focused beams of photons, electrons, or ions. In this work, we provide a solution toward wafer-scale, arbitrary modulation of feature size distribution by introducing a lithographic portfolio combining interference lithography (IL) and grayscale-patterned secondary exposure (SE). Employed after the high-throughput IL, a SE with patterned intensity distribution spatially modulates the dimensions of photoresist nanostructures. Based on this approach, we successfully fabricated 4-inch wafer-scale nanogratings with uniform linewidths of <5% variation, using grayscale-patterned SE to compensate for the linewidth difference caused by the Gaussian distribution of the laser beams in the IL. Besides, we also demonstrated a wafer-scale structural color painting by spatially modulating the filling ratio to achieve gradient grayscale color using SE.

## Abstract (web summary)

Wafer-scale fabrication of periodic nanostructures with spatially modulated feature sizes is achieved by combining interference lithography and grayscale-patterned secondary exposure, opening new pathways toward large-area nanophotonic devices.

## Graphical abstract

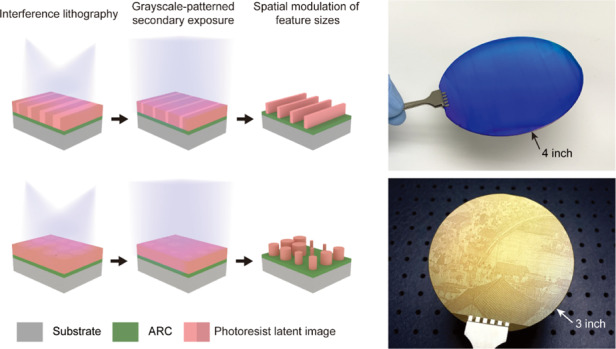

## Introduction

The precise control of feature size exhibits great importance in fabricating nanodevices for optoelectronics^1,2^, plasmonics^3,4^, metasurfaces^5,6^, and biosciences^7,8^, just to name a few. Some applications highly require uniform critical dimensions of nanostructures such as nanogratings applied in astronomy^9^, precision measurements^10^, or laser applications^11^, of which non-uniform feature sizes are regarded as defects or aberrations, deteriorating the device performance. But some devices heavily rely on nanostructures with spatially varying feature sizes, such as structural color paintings^12,13^, metalenses^14–16^, and diffractive waveguides^17,18^. For example, functional nanostructures can directly generate a structural color by separating light via scattering and diffraction, while the color is mainly dependent on the dimensions and materials^19–21^. Moreover, spatially arranging the dimensions and orientations of nanostructures enables the manipulation of light propagation via phase control, which has been widely adopted in the emerging metalenses^22,23^.

To date, the precise manipulation of feature size for nanopatterning on a large area is still challenging. Generally, the mainstream approaches for accurate nanopatterning are electron beam lithography (EBL)^24,25^ and focused ion beam (FIB) milling^26,27^. However, both are serial pixel-by-pixel writing processes, placing a limitation on the patterning area and suffering from low writing efficiency. Although high-throughput nanofabrication techniques such as interference lithography (IL)^28,29^ and nanoimprint lithography^30,31^ are capable of large-area patterning, the former is mainly for fabricating periodic structures and the latter heavily relies on the master imprint mold. In our previous work, the Gaussian-distributed beam profile in the IL has been utilized to fabricate spatially varying nanostructures with a circular gradient profile and demonstrate their applications in wettability manipulation and biosensing^32,33^. However, because the circular gradient profile is due to the Gaussian distribution of the beam intensity, it is still impossible to achieve an arbitrary modulation profile of the nanostructure feature sizes. Therefore, devising a nanopatterning approach that simultaneously satisfies (1) high-throughput and large-area patterning and (2) precisely and spatially modulating feature size, remains a challenge.

In this paper, we propose a nanopatterning strategy that combines IL and grayscale-patterned secondary exposure (IL-GPSE) to address the above challenges. Used for high-throughput fabrication of nanostructures, IL first efficiently exposes a large-area periodic nanoscale pattern on the photoresist. Then a secondary exposure (SE) of ultraviolet (UV) light, carrying a designed intensity distribution of a grayscale pattern, is applied to the IL-exposed substrate to spatially modulate the feature sizes of individual nanostructures. IL-GPSE allows wafer-scale nanostructure patterning with improved uniformity by compensating the linewidth variation caused by the non-uniform IL exposure field using a specially designed SE intensity distribution. We demonstrated a 4-inch wafer patterned by uniform 400-nm-period and 125-nm-linewidth nanogratings, with the linewidth uniformity improved by 1100% over IL-only exposure. Besides, a 3-inch grayscale painting was also demonstrated as the IL-GPSE enables precise grayscale control by tuning the filling ratio of nanostructures, indicating potential applications in flat optical devices and structural color encryption, etc.

## Results

### Mechanism of feature size modulation on the IL-exposed nanopatterns using SE

**Fig. 1:**
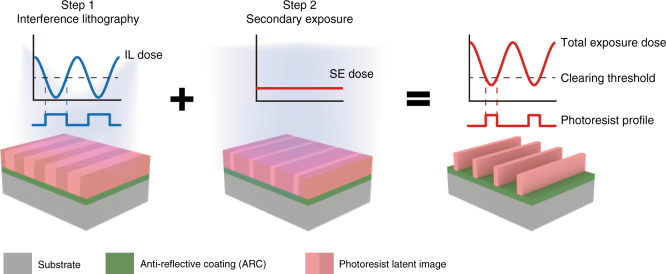
The schematic diagram of the IL-GPSE process. Step 1, a two-beam IL is performed on the photoresist to form periodic latent exposed regions with sinusoidal dose profile. Step 2, a secondary exposure follows the IL exposure and increases the effective dose applied to the photoresist which increases the portion of photoresist that receives above-threshold dose, and then leaves less photoresist after development

The feature size modulation using IL-GPSE was achieved by a combination of two exposure processes, a high-contrast IL for fabricating large-area periodic nanostructures and a SE for locally tailoring structural dimensions, as illustrated in Fig. 1. Using a positive photoresist as the example, a typical two-beam IL is first implemented to form periodic latent exposed regions with sinusoidal dose profile^34,35^. The positive photoresist that receives exposure dose above the clearing threshold would be “washed out” during the development while that below the threshold remains^36,37^. The linewidth of the developed gratings mainly depends on the area proportion exposed below the threshold^38,39^. In the IL-GPSE fabrication, a SE will follow the IL exposure and provide an additional dose that superimposes on the IL sinusoidal dose profile to increase the effective dose applied on the photoresist, therefore increasing the portion of the photoresist that receives the above-threshold dose and leaving less photoresist after development. Thus, a linewidth modulation can be achieved by superimposing the IL exposure dose with a patterned SE dose.

**Fig. 2:**
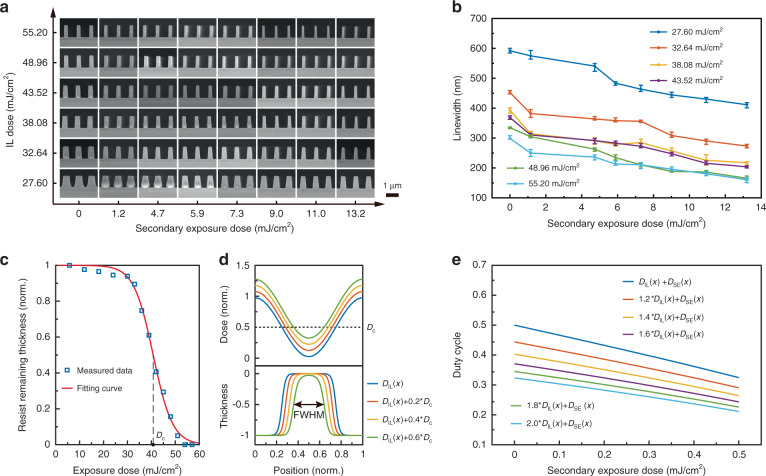
Experimental results and numerical model of feature size modulation by SE. **a** SEM image matrix of 1000-nm-period gratings with various linewidths fabricated by different IL and secondary exposure doses. **b** The FWHM linewidths measured in **a**, showing the linewidth is negatively correlated to both IL and secondary exposure doses. **c** Normalized measured photoresist remaining thickness as a function of exposure dose (blue squares) and the corresponding best-fitting curve (red line). **d** Simulation of the total exposure dose distribution and resulting developed photoresist profiles by setting photoresist contrast as extracted from **c** and contrast of IL exposure dose distribution as 0.95. **e** The simulated duty cycle variation as a function of the secondary exposure dose using various initial IL exposure doses

To demonstrate the linewidth tunability of the above lithographic strategy, a series nanogratings with various linewidths were fabricated through the IL-GPSE with different combinations of IL dose and SE dose. The samples were first exposed with 1000-nm-period grating patterns with various IL doses from 27.6 to 55.2 mJ/cm^2^, and then followed by the SE of eight flood doses ranging from 0 to 13.2 mJ/cm^2^, respectively. Figure 2a shows the SEM characterization of these nanogratings, demonstrating a reliable control of the feature sizes by modulating both the initial IL and SE doses. The full width at half-maximum (FWHM) linewidth of the nanogratings was quantitatively characterized and recorded with the corresponding exposure doses in Fig. 2b, indicating that the samples exposed by different initial IL doses show different linewidth modulation ranges during SE. For example, the samples of 1000-nm-period gratings exposed by 27.6 mJ/cm^2^ IL dose have a 180-nm modulation range of the linewidth from 590 to 410 nm, while that of 55.2 mJ/cm^2^ IL dose can be tuned with a 140-nm range from 300 to 160 nm. Another notable phenomenon is that nanogratings exposed to different exposure combinations may have a similar FWHM linewidth, however, the sidewall morphologies show a great difference since increasing IL dose benefits vertical sidewall while the SE dose has less effect. Although in this work our investigation is mainly based on positive-tone photoresist, it is worth noting that the IL-GPSE process can also apply to negative-tone photoresist (Fig. S1).

Generally, the profile of the final exposed nanostructures depends on two important factors, the photoresist contrast and the distribution of the exposure dose. The former relates to not only the resist’s material properties but also baking and development conditions, and can be experimentally characterized using the photoresist characteristic curve^40^, as shown in Fig. 2c reveals the relation between the normalized photoresist remaining thickness and the exposure dose (details in Methods). The slope of the transition region in the curve largely determines the sidewall angle of the gratings. On the other hand, the distribution of the exposure dose, particularly its contrast, as defined as the relative ratio between the local doses in the constructive and destructive interference regions, also shows a significant influence on the pattern morphology. The dose distribution in the IL exposure can be numerically modeled from the spatial distribution of the electrical field of the two interfering coherent laser beams, by taking into account the fringe drifting due to environmental disturbance^35^ (details in Methods). The total exposure dose distribution is the summation of the IL exposure dose and the SE dose. The actual nanostructure pattern can be simulated by combining the experimental photoresist characteristic curve in Fig. 2c and the total exposure dose distribution, enabling the morphology prediction and process analysis in our IL-GPSE method. Figure 2d demonstrates the simulated photoresist morphologies varied with different SE doses from 0 to 0.6*D_c_, where *D*_c_ represents the clearing dose used in the simulation for convenience (details in Methods). The FWHM linewidth shrinks as the SE dose increases, which conceptually confirms the linewidth modulation achieved by SE after IL. In addition, when assigning different photoresist contrast characteristics in the model to simulate the morphological variation, the sidewall angle changes with the photoresist contrast and it is roughly maintained during the linewidth tailoring by SE (Fig. S2). Similarly, simulations with different interference exposure dose distributions in Fig. S3 demonstrate that a high contrast in the interference exposure significantly enlarges the process window of the linewidth tunability since a low-contrast exposure results in photoresist height loss. Using this numerical model, we can also simulate the tuning range of the linewidth or the duty cycle with various initial IL exposure doses, as shown in Fig. 2e, which exhibit the similar trend as in the experimental measurement in Fig. 2b.

### Spatial modulation of nanostructure feature sizes using grayscale-patterned SE

**Fig. 3:**
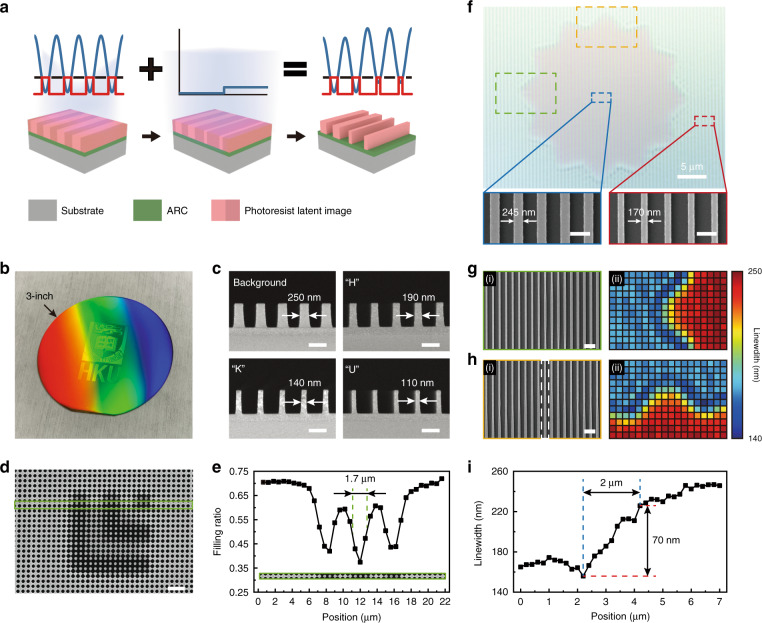
Spatial modulation of nanostructure feature sizes using grayscale-patterned SE. **a** Schematic of spatial modulation of linewidth using patterned secondary exposure. **b** A 3-inch wafer carrying the HKU logo consisting of 600-nm-period photoresist gratings with the linewidths in different regions modulated by projected grayscale pattern. **c** SEM images taken on background, “H”, “K”, and “U” letters on the wafer, showing corresponding linewidths of 250, 190, 140, and 110 nm. **d** SEM image of a 600-nm-period hole array modulated by DLW secondary exposure. **e** Plot of the filling ratio of nanostructures in the green box in **d** versus the horizontal position, showing a resolution of 1.7 µm. **f** Optical microscope image of a 12-point-star pattern, consisting of 600-nm-period gratings with linewidths modulated by contact photolithography using a photomask. SEM images taken inside and outside the secondary exposure pattern, showing the 245-nm and 170-nm linewidths, respectively. **g** Green box and **h** yellow-box regions in **f**, characterized by (i) SEM images and (ii) linewidth distributions. **i** The linewidth variation measured every 200 nm along a grating line (white, Fig. 3h), showing the grating linewidth gradient of 35 nm/µm. Scale bars, 500 nm (**c**, **f**), 1 µm (**g**, **h**), and 2 µm (**d**)

The linewidth manipulation mechanism makes it possible to modulate the nanostructure feature sizes according to a given spatial pattern (Fig. 3a). The patterned SE can be realized through a UV projector, a UV mask aligner, or direct laser writing (DLW). For example, a UV projector is capable of converting a digital grayscale image into the UV intensity distribution for spatially modulating the dimensions (Fig. S4). Figure 3b shows a 3-inch Si wafer carrying an HKU logo, consisting of 600-nm-period photoresist gratings with the linewidths in different regions modulated by the projected grayscale pattern. The photoresist was first exposed with 600-nm-period gratings using IL and then a grayscale image of an HKU logo was projected through a 405-nm projector (Fig. S5). The grayscale pattern projected on the pre-exposed photoresist spatially modulates the linewidths according to the digital grayscale image. SEM images were taken on different parts of the image, including the areas of background, “H”, “K”, and “U” letters respectively, demonstrating the corresponding linewidths of 250, 190, 140, and 110 nm, which indicates a >55% linewidth modulation range (Fig. 3c).

Our proposed strategy allows feature size tuning at micrometer scale, subject to the spatial resolution of the grayscale image of the patterned SE. As IL exposure creates a nearly uniform or slowly varying latent dose image in the photoresist, the resolution of the feature size modulation mainly depends on the resolution of the SE. We investigated the spatial resolution of SE using DLW at different critical dimensions (Fig. S6). The SEM image in Fig. 3d shows an IL-exposed nanohole array in which some holes are transformed into pillars with a SE modulation. The filling ratio of the modulated patterns ranges from over 0.7 to below 0.4 and demonstrates a spatial resolution of ~1.7 microns, as illustrated in Fig. 3e. With single-pixel exposure of 1-µm resolution, we further achieved the fabrication of a photonic crystal pattern with a defect, providing an innovative way to fabricate photonic crystal cavities over a large area for fundamental photonics research and practical device applications, as shown in Fig. S7.

In addition, we also carried out the linewidth modulation on a uniform nanograting using contact photolithography with a photomask. The optical microscope image in Fig. 3f shows a 12-point-star pattern consisting of 600-nm-period gratings with 245-nm linewidth inside and 170-nm outside. Figure 3g(i) and 3h(i) show the SEM images of the photoresist gratings recorded in the tip regions, labeled using green and yellow boxes, in which the grating linewidth changes within a short distance at the pattern edge. The linewidth distribution is mapped in Fig. 3g(ii) and 3h(ii) clearly exhibited the tip outlines with a transition distance of ~2 microns, as quantitatively analyzed in Fig. 3i. The precise quantitative characterization by measuring the linewidth every 200 nm along a grating line (white, Fig. 3h) shows a 35-nm/µm grating linewidth gradient induced by the edge diffraction of patterned SE.

### Uniform 4-inch wafer-scale patterning using IL-GPSE

**Fig. 4:**
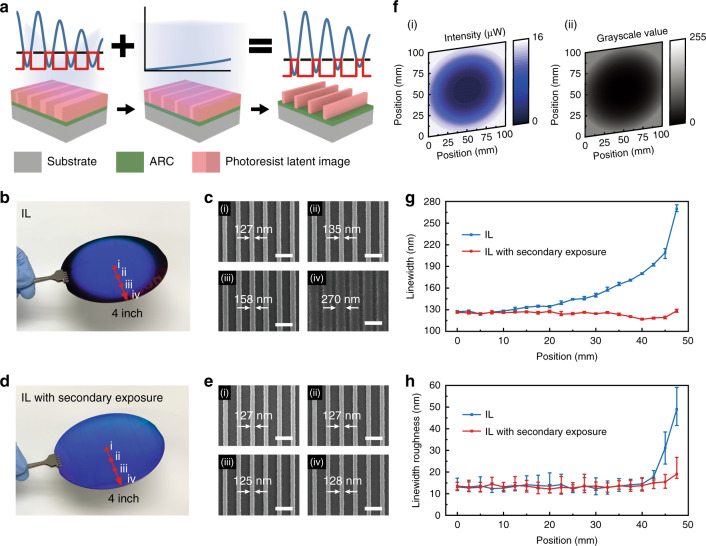
Uniform 4-inch wafer-scale patterning using IL-GPSE. **a** Schematic of uniform nanopatterning using secondary exposure to compensate the linewidth variation due to the non-uniform laser beam intensity during IL. **b** Photograph of IL-exposed 400-nm-period gratings over a 4-inch wafer with the diffracted blue light intensity gradually decreasing from the center to the edge. **c** SEM images taken in the labeled positions in **b**, showing a poor control of linewidth and roughness. **d** Photograph of 400-nm-period gratings over a 4-inch wafer using secondary exposure with a designed projected grayscale pattern after IL exposure, diffracting uniform blue light. **e** SEM images recorded in the labeled positions in **g**, showing the uniform linewidth and linewidth roughness. **f** The 4-inch (i) intensity distribution for linewidth tailoring and (ii) devised grayscale image transformed by the characterized relation between the digital grayscale value and projected light intensity via the UV projector. The comparisons of **g** linewidth and **h** linewidth roughness distributions over the radius of 4-inch wafers w/o the secondary exposure, respectively. Scale bars: 500 nm

The proposed IL-GPSE can also be applied on non-uniform nanostructures, which are caused by the non-uniform laser beam intensity during IL, to compensate for the spatial variation for improved uniformity, which is essential in many optical applications that demand large-area uniform gratings^41,42^ and precise interferometry^43^. Generally, large-area nanogratings fabricated by IL suffer radial-gradient linewidths due to the Gaussian profile in the interfering beams, and researchers often expand the Gaussian beam^44^ or implement beam shaping devices to transform the Gaussian distribution to a flat top distribution^45^ to acquire a relatively uniform intensity at the central exposure area. However, the former wastes a large amount of exposure energy and decreases the production capacity while the latter introduces a new challenge of fabricating high-quality large-area beam shaping devices. Employing the SE with a properly designed intensity distribution after the conventional IL with Gaussian intensity profile offers a new and efficient approach to compensating the linewidth variation (Fig. 4a). Figure 4b shows IL-exposed 400-nm-period gratings over a 4-inch wafer. The diffracted blue light intensity gradually decreases from the center to the edge due to Gaussian intensity distribution (Fig. S8), resulting in an increasing linewidth variation from the center to the edge due to the spatially decreasing intensity. We recorded SEM images every 2.5 mm along the radius of the 4-inch wafer (Fig. S9), morphologies of four typical positions were illustrated in Fig. 4c to show the linewidths of 127, 135, 158, and 270 nm, of which a poor control of both linewidth and linewidth roughness was observed near the edge.

In contrast, by performing the SE with a designed intensity distribution complementary to the Gaussian distribution, the pattern uniformity, and line edge roughness can be significantly improved. By referring to SEM images and corresponding linewidths under different IL and SE doses, as shown in Figs. S10 and S11, respectively, the SE intensity map required for compensating the non-uniform IL linewidth variation on the 4-inch wafer can be designed. Figure 4f shows the 4-inch intensity map for linewidth tailoring and the devised grayscale image, transformed by using the characterized relationships between the digital grayscale value and projected light intensity via the UV projector (Fig. S12). After applying the SE with the projected grayscale pattern, a 4-inch wafer carrying gratings with highly homogeneous linewidths was obtained and uniformly diffracted blue light, as shown in Fig. 4d. SEM images were obtained on both un-compensated and compensated grating samples for comparison, as in Fig. 4c, e, and showed a significant improvement in grating linewidth uniformity and line edge roughness.

The linewidth distributions were statistically analyzed by measuring the linewidth of the SEM image captured every 2.5 mm along the radius from the wafer center to the edge (Fig. S13). The comparison of linewidth distributions in Fig. 4g shows that gratings tailored by SE had a linewidth deviation of 3.2 nm, which is over 1100% reduced from 36.2 nm, that of the gratings exposed only by IL. The SEM images also show that the linewidth roughness was significantly improved through SE, especially for the gratings near the edge. The difference originates from the total exposure dose on the photoresist, of which a higher dose facilitates the diffusion of photoacid compounds. As a result, a >580% improvement (from 8.7 nm to 1.5 nm) is obtained in the linewidth roughness characterization when the SE is employed (Fig. 4h).

### Large-area grayscale painting using IL-GPSE

**Fig. 5:**
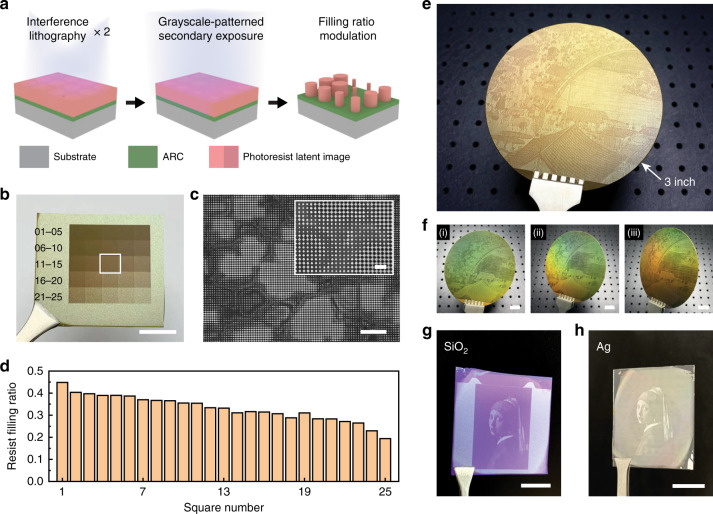
Large-area grayscale painting using IL-GPSE. **a** Schematic of spatial modulation of filling ratio for 2D nanostructures using IL-GPSE. **b** The photograph of 25 squares of 700-nm-period 2D patterns on photoresist modulated by SE projection, showing a clear grayscale change from dark brown to light gold. **c** SEM images of the region labeled using white box in **b**. **d** Fraction area of photoresist calculated from SEM images of Fig. S15, showing a negative correlation between the photoresist filling ratio and the SE dose. **e** A photograph of the 3-inch grayscale painting of *Along the River During the Qingming Festival* in photoresist patterned by IL-GPSE. **f** A dynamic structural color due to the residual diffraction at tilting angles. Grayscale paintings patterned by IL-GPSE are further **g** etched into 300-nm-thick SiO_2_-coated Si wafer, or **h** deposited with 50-nm-thick Ag on glass. Scale bars, 2 µm (inset in **c**), 10 µm (**c**), and 1 cm (**b**, **f**–**h**)

In addition to the large-area patterning of uniform gratings, the IL-GPSE method also enables the filling ratio modulation for two-dimensional (2D) nanostructures (Fig. 5a), which are major building blocks in structural color based paintings^46^. Most structural color paintings using 2D nanostructures can be fabricated only on small areas of millimeter scale or less because they need to use EBL, an expensive and time-consuming tool, for high-resolution patterning of spatially varying structures. By adopting our proposed IL-GPSE method, structural color paintings can be fabricated on wafer-scale, because both the IL and patterned SE can be done at a large scale while maintaining sufficiently high resolution. Another factor that needs to be considered is that the diffraction-induced coloration caused by the nanostructure periodicity will interfere with the designed structural color, especially over large-area samples^47,48^. We intentionally adopt a microscopically roughened surface to mitigate this diffraction effect by randomly scattering the diffracted light. We applied the IL-GPSE process on the unpolished side of an Si wafer and investigated the structural color variation under various SE intensities. As shown in Fig. 5b, 25 squares of 700-nm-period 2D patterns on photoresist modulated by SE projection show a clear grayscale color change from dark brown to light gold, of which the corresponding reflectance increases (Fig. S14). SEM characterization shows non-uniform 2D periodic nanopatterns on the microscopically irregular surface (Fig. 5c and Fig. S15), from which the filling fraction of the photoresist can be extracted (details in Methods). Figure 5d demonstrates that the photoresist filling ratio is negatively correlated to the SE dose, which confirms the lateral filling ratio modulation by SE enables the grayscale tunability.

We further demonstrate a grayscale painting using wafer-scale IL-GPSE. The SE projects part of the renowned Chinese artistic painting *Along the River During the Qingming Festival* (Chinese name: *Qingming Shanghe Tu*) by Zhang Zeduan (1085–1145) (Fig. S16) on a 3-inch wafer, consisting of a latent image of 700-nm-period 2D patterns in the photoresist. After photoresist development, a grayscale replication of the painting appears on the Si substrate (Fig. 5e). When the wafer is tilted at different angles, a dynamic structural color due to the residual diffraction can also be observed (Fig. 5f), indicating potential applications in cryptography or anti-counterfeiting. Moreover, pattern transfer into other materials can be realized by combining IL-GPSE strategy with other nanofabrication techniques such as reactive ion etching and electron beam evaporation. As an example, Fig. 5g,h show the grayscale paintings of *Girl with a Pearl Earring* engraved in a 300-nm-thick SiO_2_-coated Si wafer and deposited with 50-nm-thick Ag on a glass substrate, respectively.

## Discussion

In summary, we have invented a practical and scalable lithographic strategy that supports spatial modulation of the feature size of periodic nanostructures on a large area. Our fabrication portfolio uses IL to efficiently fabricate large-area periodic nanostructures and grayscale-patterned SE to spatially modulate the feature sizes. We successfully fabricated highly uniform nanogratings on a 4-inch wafer by compensating for the effect of the non-uniform Gaussian profile of the interference laser beams using a grayscale-patterned SE. Additionally, we demonstrated 3-inch grayscale painting with 2D nanostructures fabricated by IL-GPSE, which may also find potential applications in information encryption, anti-counterfeiting, etc. Our method can also benefit the fabrication of metasurfaces and metalenses that employ nanostructures with spatially modulated filling ratios^49^. By separating the high-resolution patterning of metasurface building blocks and the size modulation, our method can improve the patterning efficiency for these devices by orders of magnitude when compared with EBL.

## Materials and methods

### Fabrication of photoresist nanogratings

Firstly, the silicon wafer was ultrasonic cleaned with acetone, isopropanol, and deionized water for 5 min, respectively. Then after oxygen plasma treatment, the Si wafer was spin-coated with a 200-nm-thick anti-reflective coating (ARC; AZ BARLi-II, MicroChemicals, GmbH) layer at 2000 rpm and baked at 200 °C for 2 min to eliminate the standing wave effect in an exposure. Then the positive photoresist (AZ MiR 701, MicroChemicals, GmbH) was spin-coated at 3000 rpm and pre-baked at 90 °C for 60 s. Original and diluted (1:1 with propylene glycol methyl ether acetate) photoresists were used for patterning the periods of 1000 nm/700 nm/600 nm and 400 nm, respectively. The samples were first exposed by a home-made IL system with a 405-nm laser (Omicron, GmbH) to form large-area periodic latent nanostructures and then were exposed via a UV projector (Xiamen Zhisen Electro. Equip. Co., Ltd.), a UV mask aligner (URE-2000/35, Chinese Academy of Sciences), or a DLW machine (MicroWriter ML3, Durham Magneto Optics Ltd.) to spatially modulate the latent feature sizes. After performing IL-GPSE process, samples were post-baked at 110 °C for 60 s, developed (AZ 726 MIF, MicroChemicals, GmbH) for 60 s, and rinsed thoroughly with deionized water.

### Obtaining the photoresist characteristic curves

The raw data were recorded by exposing 800-nm-thick photoresist on ARC/Si substrates with different exposure doses and then measuring the corresponding residual thicknesses after development. The remaining photoresist thickness, *T*, was then normalized to the initial unexposed thickness and plotted as a fitting function of the dose, *D*, to fit the characteristic curve^50^:

1 $$T\left( D \right) = \frac{1}{{1 + e^{{\gamma }\left( {D - D_{{{{\mathrm{c}}}}}}\right)}}}$$

where *D*_c_ is the clearing dose and γ is the photoresist contrast, extracted to be 40.77 and 0.2474. As a convenience, *D*_c_ and γ are regarded as basic units of parameters in simulations. Notably, the photoresist characteristic curves should be recalibrated with experimental data when the process conditions, such as the photoresist type, thickness, development parameters, etc., are changed.

### Numerical model of IL-GPSE strategy

The interference exposure dose distribution on the photoresist can be calculated as

2 $$D_{{{{\mathrm{IL}}}}}\left( x \right) = D_{{{{\mathrm{IL}}}}}\left[ {1 + {\Gamma}\cos \left( {\frac{{2\pi x}}{p}} \right)} \right]$$

where Γ is the contrast of the interference exposure dose distribution, *p* is the grating period, and *x* is the horizontal position. By superimposing a SE on the IL dose distribution, the total exposure dose can be described as

3 $$D_{{{\mathrm{T}}}}\left( x \right) = D_{{{{\mathrm{IL}}}}}\left( x \right) + D_{{{{\mathrm{SE}}}}}\left( x \right)$$

where *D*_SE_(*x*) is the distribution of SE dose. When the SE is a flood exposure, *D*_SE_(*x*) should be a constant; when it is a grayscale pattern exposure, *D*_SE_(*x*) should be a variable depending on spatial distribution. By applying Eqs. (1–3), a spatial photoresist thickness variation can be obtained as the numerical model.

### Extraction of the photoresist filling ratio

SEM images are saved as uint16 data type which shows morphological information of nanostructures using 256 grayscale values. In the SEM images, the photoresist appears brighter while the exposed silicon surface appears dark. By obtaining the grayscale values of all pixels and setting a properly chosen threshold, the number of pixels that are covered by photoresist can be counted and the photoresist filling ratio can be calculated.

### Fabrication of the grayscale painting

For the grayscale painting of *Along the River During the Qingming Festival*, IL-GPSE was employed on the backside of a 3-inch 200-nm-thick SiO_2_-coated silicon wafer, spin-coated with 200-nm-thick ARC and 800-nm-thick photoresist. For the grayscale painting of *Girl with a Pearl Earring* in Fig. 5g, IL-GPSE was first employed on the backside of a 300-nm-thick SiO_2_-coated silicon substrate, spin-coated with 200-nm-thick ARC and 800-nm-thick photoresist. After patterning on the photoresist, the underlying ARC and SiO_2_ layers were etched using O_2_ plasma and CHF_3_/H_2_ in a reactive ion etching system (RIE-10NR, Samco), respectively, to transfer the pattern into SiO_2_. Finally, the photoresist and ARC residues were removed to complete the grayscale painting. For the grayscale painting of *Girl with a Pearl Earring* in Fig. 5h, IL-GPSE was first employed on a glass substrate, spin-coated with 200-nm-thick ARC and 800-nm-thick photoresist. After patterning in the photoresist, the underlying ARC layer was etched using O_2_ plasma to expose the surface of glass. Next, 50-nm-thick Ag was deposited using electron beam evaporation (EB500-I, Shenyang Scientific Instrument Ltd.). Finally, a lift-off process was performed with ultrasonic cleaning in RCA-1 solution (NH_3_·H_2_O:H_2_O_2_:H_2_O = 1:1:5) at 80 °C for 3 min, leaving the glass substrate with Ag grayscale pattern.

### Characterizations

SEM (Zeiss Sigma 300) was performed to characterize the morphologies of the nanostructures. SEM images were analyzed by a commercial software ProSEM (GenISys Ltd.) statistically calculate the linewidth and roughness. The photoresist thickness was measured by an ellipsometer (TF-UVISEL, HORIBA Jobin Yvon). The light intensity was measured by a commercial digital power meter (PM160, Thorlabs, Inc.). The reflective spectra were collected using a commercial spectrometer (QE65Pro, Ocean Optics).

## Supplementary information

Supplementary Information

## References

[1] Chen LY (2019). Nanostructured texturing of CH_3_NH_3_PbI_3_ perovskite thin film on flexible substrate for photodetector application. Org. Electron..

[2] Joo WJ (2020). Metasurface-driven OLED displays beyond 10,000 pixels per inch. Science.

[3] Li WD (2011). Three-dimensional cavity nanoantenna coupled plasmonic nanodots for ultrahigh and uniform surface-enhanced Raman scattering over large area. Opt. Express.

[4] Cai JX (2019). Highly-facile template-based selective electroless metallization of micro- and nanopatterns for plastic electronics and plasmonics. J. Mater. Chem. C..

[5] Khorasaninejad M (2016). Metalenses at visible wavelengths: diffraction-limited focusing and subwavelength resolution imaging. Science.

[6] Zheng HY (2021). Large-scale metasurfaces based on grayscale nanosphere lithography. ACS Photonics.

[7] Cai JX (2019). Solution-processed large-area gold nanocheckerboard metasurfaces on flexible plastics for plasmonic biomolecular sensing. Adv. Optical Mater..

[8] Im H (2014). Label-free detection and molecular profiling of exosomes with a nano-plasmonic sensor. Nat. Biotechnol..

[9] Savas TA (1996). Large-area achromatic interferometric lithography for 100 nm period gratings and grids. J. Vac. Sci. Technol..

[10] Hu PC (2019). Displacement measuring grating interferometer: a review. Front. Inf. Technol. Electron. Eng..

[11] Kang MS, Han C, Jeon H (2020). Submicrometer-scale pattern generation via maskless digital photolithography. Optica.

[12] Dong ZG (2017). Printing beyond sRGB color gamut by mimicking silicon nanostructures in free-space. Nano Lett..

[13] Wang H (2021). Full color and grayscale painting with 3D printed low-index nanopillars. Nano Lett..

[14] Khorasaninejad M, Capasso F (2017). Metalenses: versatile multifunctional photonic components. Science.

[15] Wang SM (2018). A broadband achromatic metalens in the visible. Nat. Nanotechnol..

[16] Lin RJ (2019). Achromatic metalens array for full-colour light-field imaging. Nat. Nanotechnol..

[17] Liu ZY (2017). Design of a uniform-illumination binocular waveguide display with diffraction gratings and freeform optics. Opt. Express.

[18] Gu L (2019). Design of a uniform-illumination two-dimensional waveguide head-up display with thin plate compensator. Opt. Express.

[19] Zhu XL (2017). Resonant laser printing of structural colors on high-index dielectric metasurfaces. Sci. Adv..

[20] Yang WH (2020). All-dielectric metasurface for high-performance structural color. Nat. Commun..

[21] Sun S (2017). All-dielectric full-color printing with TiO_2_ metasurfaces. ACS Nano.

[22] Yu NF (2011). Light propagation with phase discontinuities: generalized laws of reflection and refraction. Science.

[23] Yoon G (2020). Single-step manufacturing of hierarchical dielectric metalens in the visible. Nat. Commun..

[24] Kumar K (2012). Printing colour at the optical diffraction limit. Nat. Nanotechnol..

[25] McDonnell C (2021). Functional THz emitters based on Pancharatnam-Berry phase nonlinear metasurfaces. Nat. Commun..

[26] Tang YT (2020). Nano-kirigami metasurface with giant nonlinear optical circular dichroism. Laser Photonics Rev..

[27] Cai JX (2018). 3D volumetric energy deposition of focused helium ion beam lithography: visualization, modeling, and applications in nanofabrication. Adv. Mater. Interfaces.

[28] Farhoud M (1999). Fabrication of 200 nm period nanomagnet arrays using interference lithography and a negative resist. J. Vac. Sci. Technol..

[29] Xia DY (2011). Nanostructures and functional materials fabricated by interferometric lithography. Adv. Mater..

[30] Chou SY, Krauss PR, Renstrom PJ (1996). Nanoimprint lithography. J. Vac. Sci. Technol..

[31] Guo LJ (2007). Nanoimprint lithography: methods and material requirements. Adv. Mater..

[32] Min SY (2020). Gradient wettability induced by deterministically patterned nanostructures. Microsyst. Nanoeng..

[33] Min SY (2021). Ultrasensitive molecular detection by imaging of centimeter-scale metasurfaces with a deterministic gradient geometry. Adv. Mater..

[34] Liang CW (2018). Wafer-scale nanopatterning using fast-reconfigurable and actively-stabilized two-beam fiber-optic interference lithography. Opt. Express.

[35] Gan ZF (2019). Patterning of high-aspect-ratio nanogratings using phase-locked two-beam fiber-optic interference lithography. J. Vac. Sci. Technol..

[36] Levinson, H. J. Principles of Lithography. (Bellingham: SPIE Press, 2005).

[37] Mack, C. Fundamental Principles of Optical Lithography: The Science of Microfabrication. (Chichester: John Wiley & Sons, 2007).

[38] O’Reilly TB, Smith HI (2008). Photoresist characterization using double exposures with interference lithography. J. Vac. Sci. Technol..

[39] Chang EC (2013). Improving feature size uniformity from interference lithography systems with non-uniform intensity profiles. Nanotechnology.

[40] Hinsberg W (1998). Deep-ultraviolet interferometric lithography as a tool for assessment of chemically amplified photoresist performance. J. Vac. Sci. Technol..

[41] Haas, M. R. Optical design and diffraction analysis for AIRES: an airborne infrared echelle spectometer. *Proceedings of SPIE 4857, Airborne Telescope Systems II*. Waikoloa, Hawai’i, United States: SPIE, 85–26 (2003).

[42] Barnes, S. I. et al. The optical design of the Southern African Large Telescope high resolution spectrograph: SALT HRS. *Proceedings of SPIE 7014, Ground-based and Airborne Instrumentation for Astronomy II*. Marseille, France: SPIE, 70140K (2008).

[43] Lu PP (2009). Precise diffraction efficiency measurements of large-area greater-than-99%-efficient dielectric gratings at the Littrow angle. Opt. Lett..

[44] Yue G (2018). Fabrication of 4-inch nano patterned wafer with high uniformity by laser interference lithography. Chin. Phys. Lett..

[45] Yang, Y. K. et al. Improve large area uniformity and production capacity of laser interference lithography with beam flattening device. *Proceedings of SPIE 9736, Laser-based Micro- and Nanoprocessing X*. San Francisco, California, United States: SPIE, 97360Y (2016).

[46] Jalali M (2016). Stacking of colors in exfoliable plasmonic superlattices. Nanoscale.

[47] Ng RJH (2020). Darkfield colors from multi-periodic arrays of gap plasmon resonators. Nanophotonics.

[48] Ruan QF (2022). Reconfiguring colors of single relief structures by directional stretching. Adv. Mater..

[49] Yoon G (2021). Printable nanocomposite metalens for high-contrast near-infrared imaging. ACS Nano.

[50] Fallica R (2017). High-resolution grayscale patterning using extreme ultraviolet interference lithography. Microelectron. Eng..

