# Supplementary material for: Spatial modulation of nanopattern dimensions by combining interference lithography and grayscale-patterned secondary exposure
Source: Light Sci Appl. 2022 Apr 8;11:89. doi: 10.1038/s41377-022-00774-z (PMC8993805; doi:10.1038/s41377-022-00774-z)
Supplement: Supplementary file 1 — Supplementary Information [file 41377_2022_774_MOESM1_ESM.docx]

*Supplementary Information for*

**Spatial Modulation of Nanopattern Dimensions by Combining** **Interference Lithography and Grayscale-Patterned** **Secondary Exposure**

Zhuofei Gan^1,2^, Hongtao Feng^1^, Liyang Chen^1^, Siyi Min^1^, Chuwei Liang^1^, Menghong Xu^1^, Zijie Jiang^1^, Zhao Sun^1^, Chuying Sun^1^, Dehu Cui^2^, and Wen-Di Li^1, *^

^1^ *Department of Mechanical Engineering, University of Hong Kong, Hong Kong, China*

^2^ *School of Microelectronics, Southern University of Science and Technology, Shenzhen, China*

*∗ Corresponding author.*

*E-mail addresses:* [*liwd@hku.hk*](mailto:liwd@hku.hk) *(W. D. Li).*


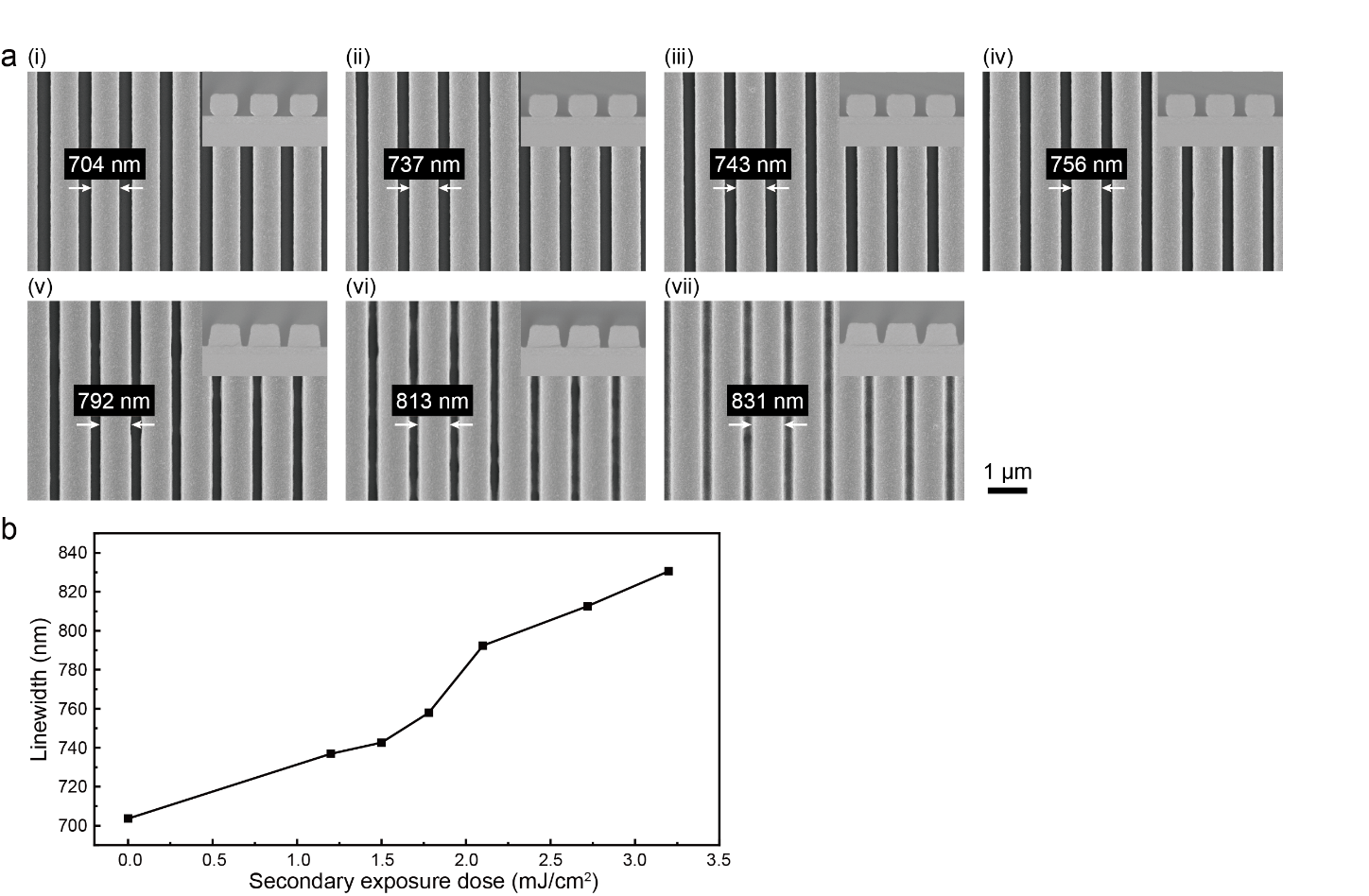


**Fig. S1.** (**a**) SEM images of 1000-nm-period gratings in negative-tone photoresist patterned by IL-GPSE process, showing the linewidth modulated from 704 to 831 nm with increasing secondary exposure dose. (**b**) The measured linewidths showing a positive correlation to secondary exposure dose.


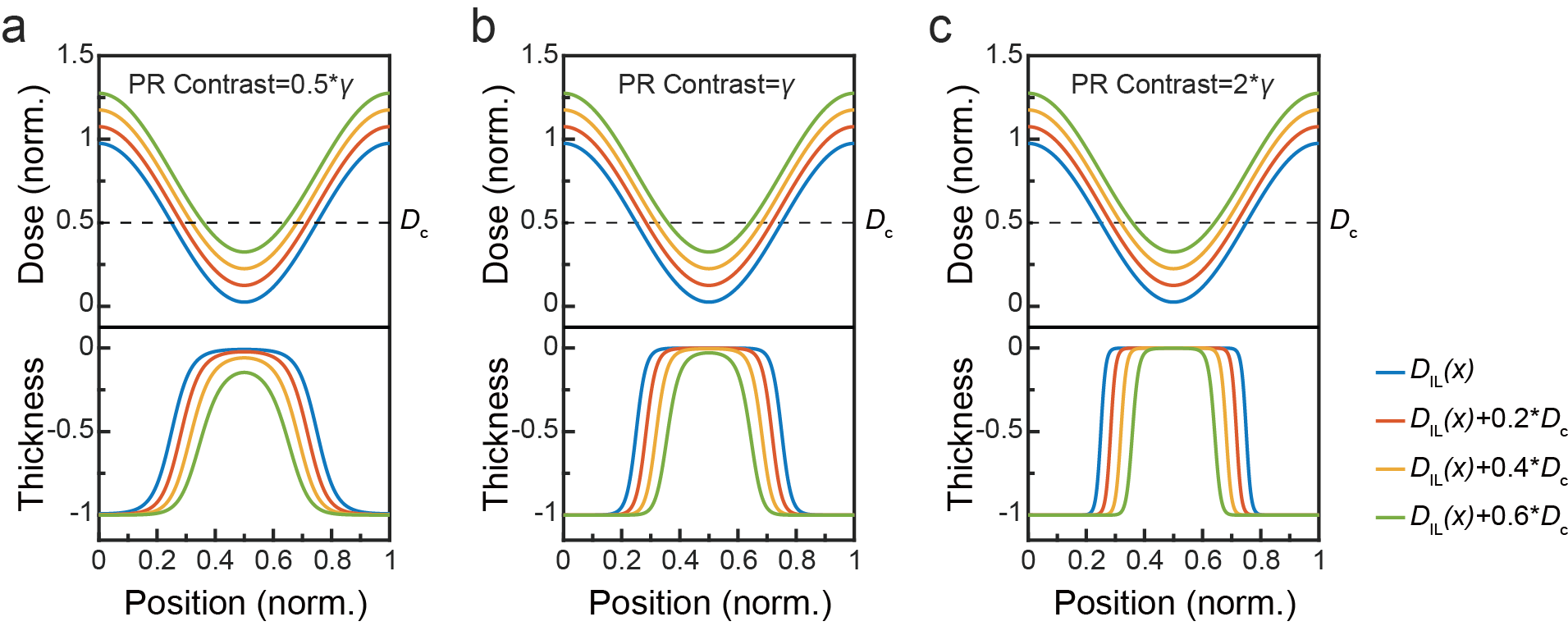


**Fig. S2.** Simulated photoresist profiles using IL-GPSE model by assigning different photoresist (PR) contrasts: (**a**) 0.5* *γ*, (**b**) *γ* and (**c**) 2* *γ*.

**
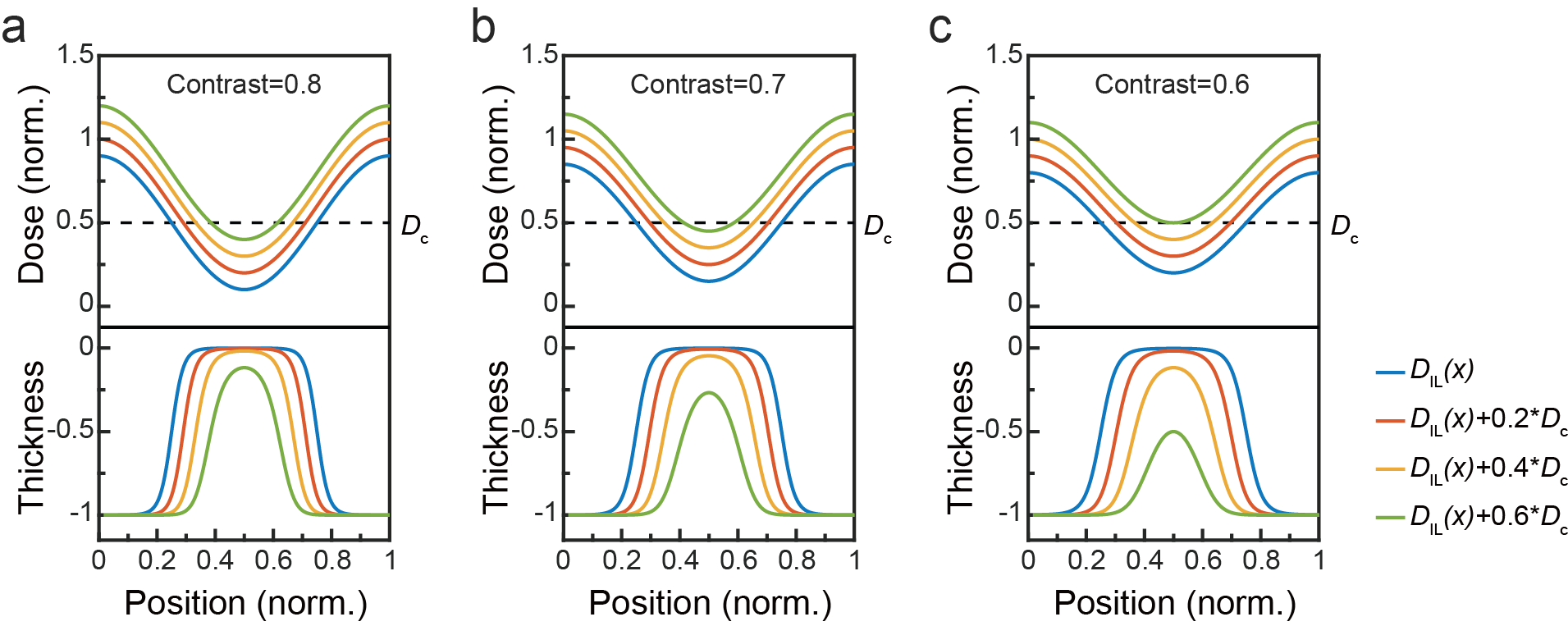
 Fig. S3.** Simulated photoresist profiles using IL-GPSE model by assigning different contrasts of the interference exposure dose distribution: (**a**) 0.8, (**b**) 0.7 and (**c**) 0.6.


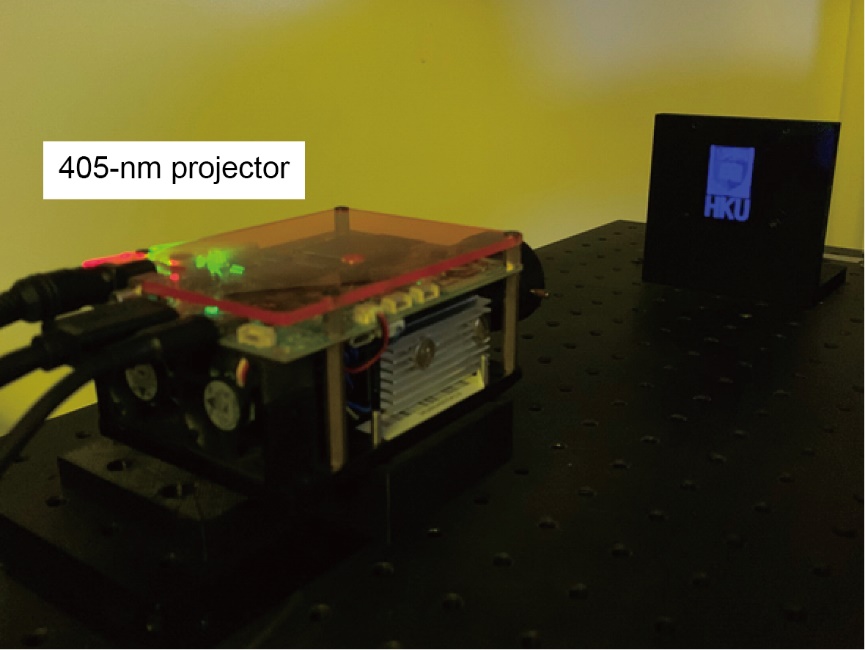


**Fig. S4.** Setup of the UV projection for secondary exposure with a digital grayscale image.


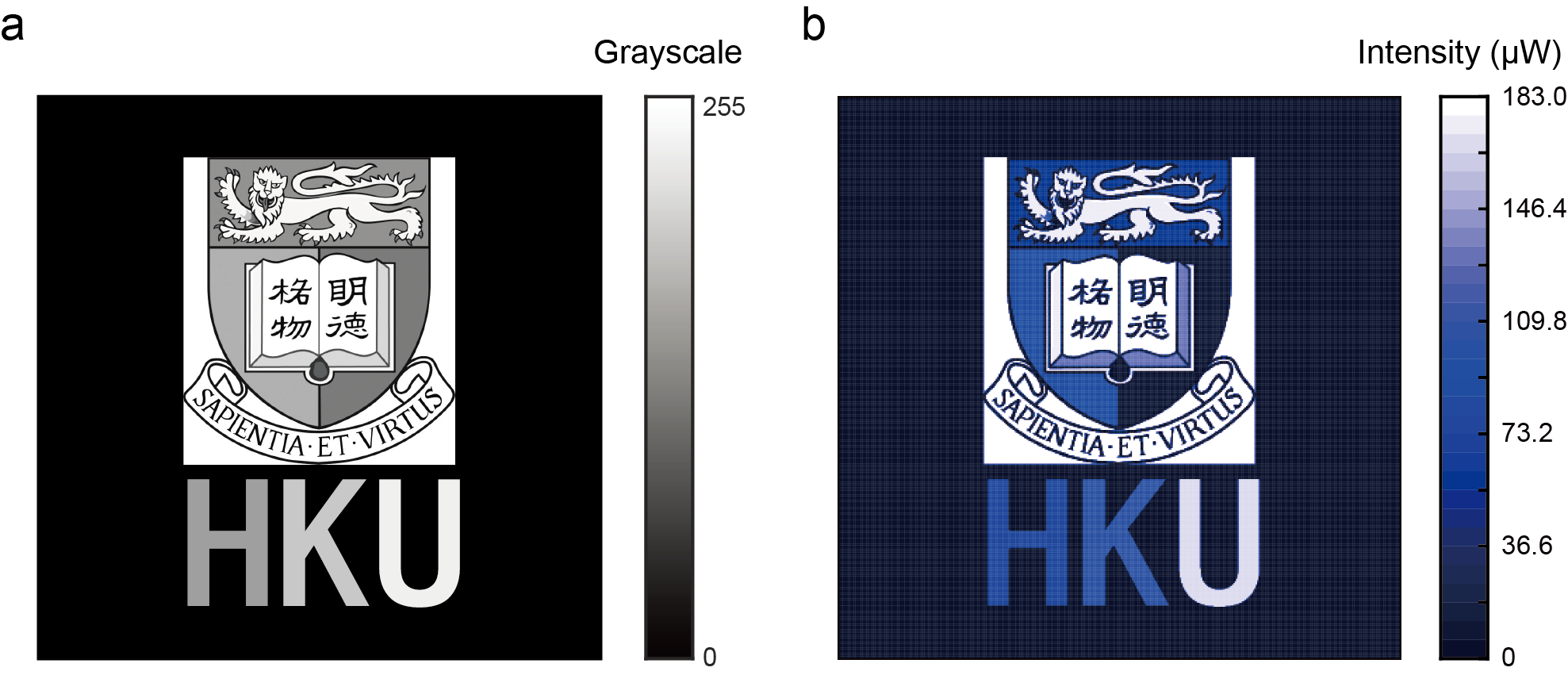
**Fig. S5.** (**a**) Digital grayscale image for the UV projection to generate a patterned UV intensity distribution. (**b**) The intensity distribution converted from (**a**) for secondary exposure.


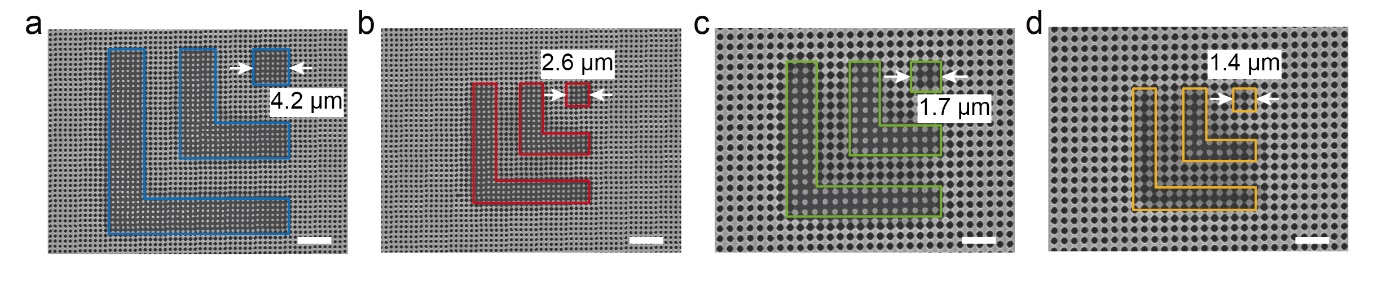


**Fig. S6.** SEM images of the spatial resolution test for secondary exposure using direct laser writing with different critical dimensions of (**a**) 4.2 μm, (**b**) 2.6 μm, (**c**) 1.7 μm and (**d**) 1.4 μm. Scale bars, 4 μm (**a, b**) and 2 μm (**c, d**).


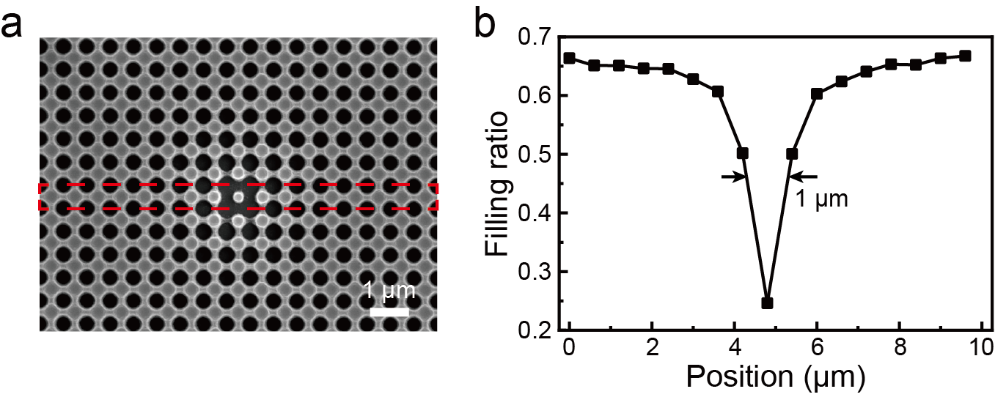


**Fig. S7.** (**a**) SEM image of a 600-nm-period hole array modulated by single-pixel secondary exposure. (**b**) Plot of the filling ratio of nanostructures in the red box in (**a**) versus the horizontal position, showing a resolution of 1 µm.


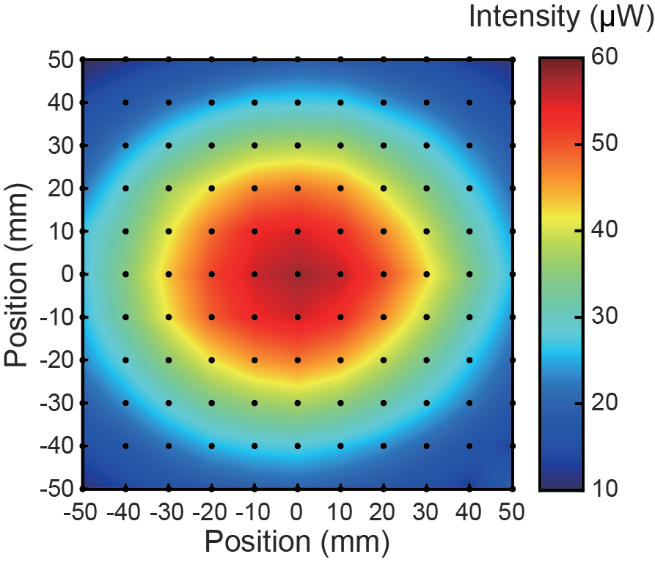


**Fig. S8.** The Gaussian distribution of the interfering beam spot measured every 1 cm on 4-inch wafer scale and plotted by interpolation fitting.


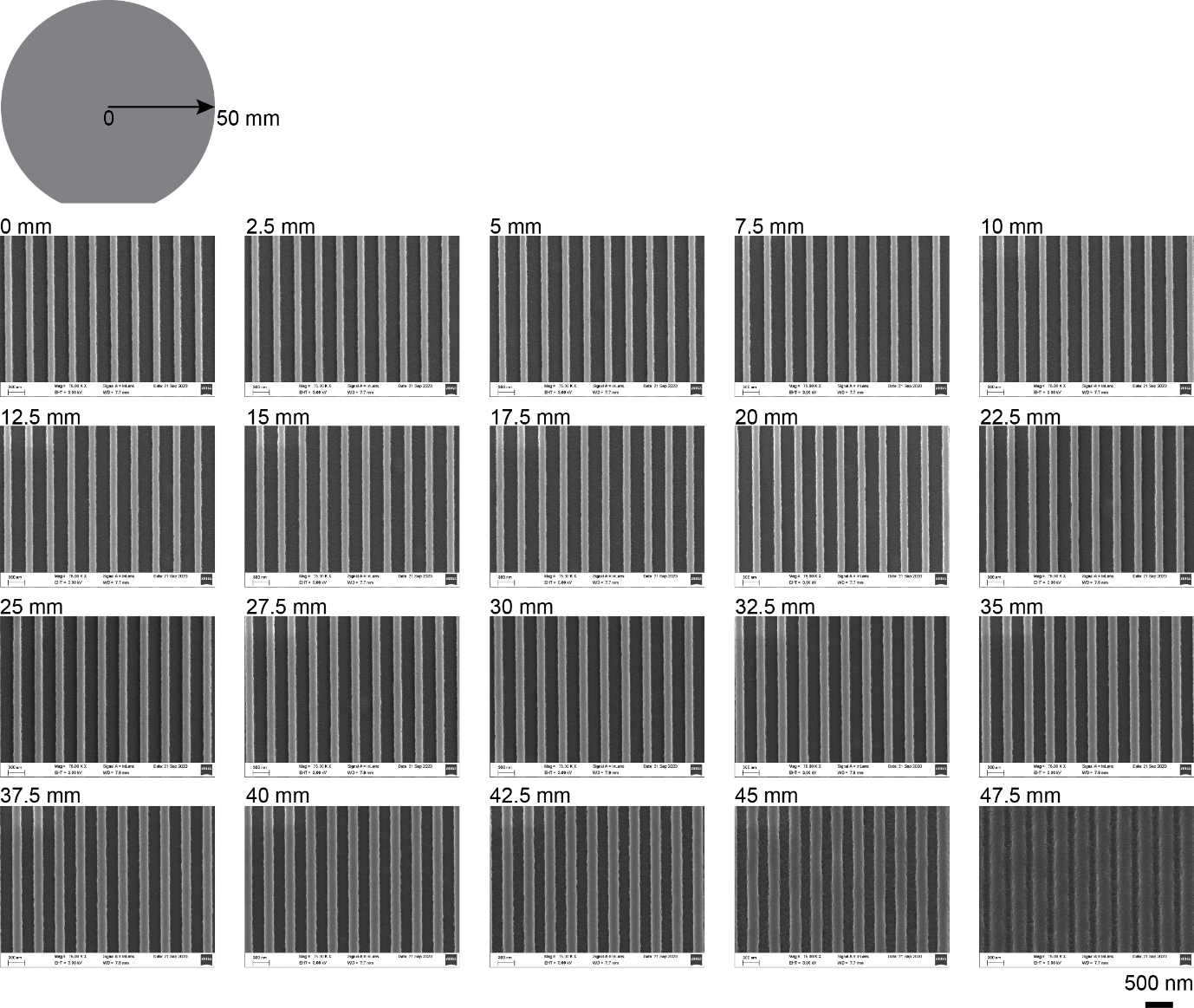


**Fig. S9.** SEM images recorded every 2.5 mm along the radius of the 4-inch wafer patterned only by interference lithography.


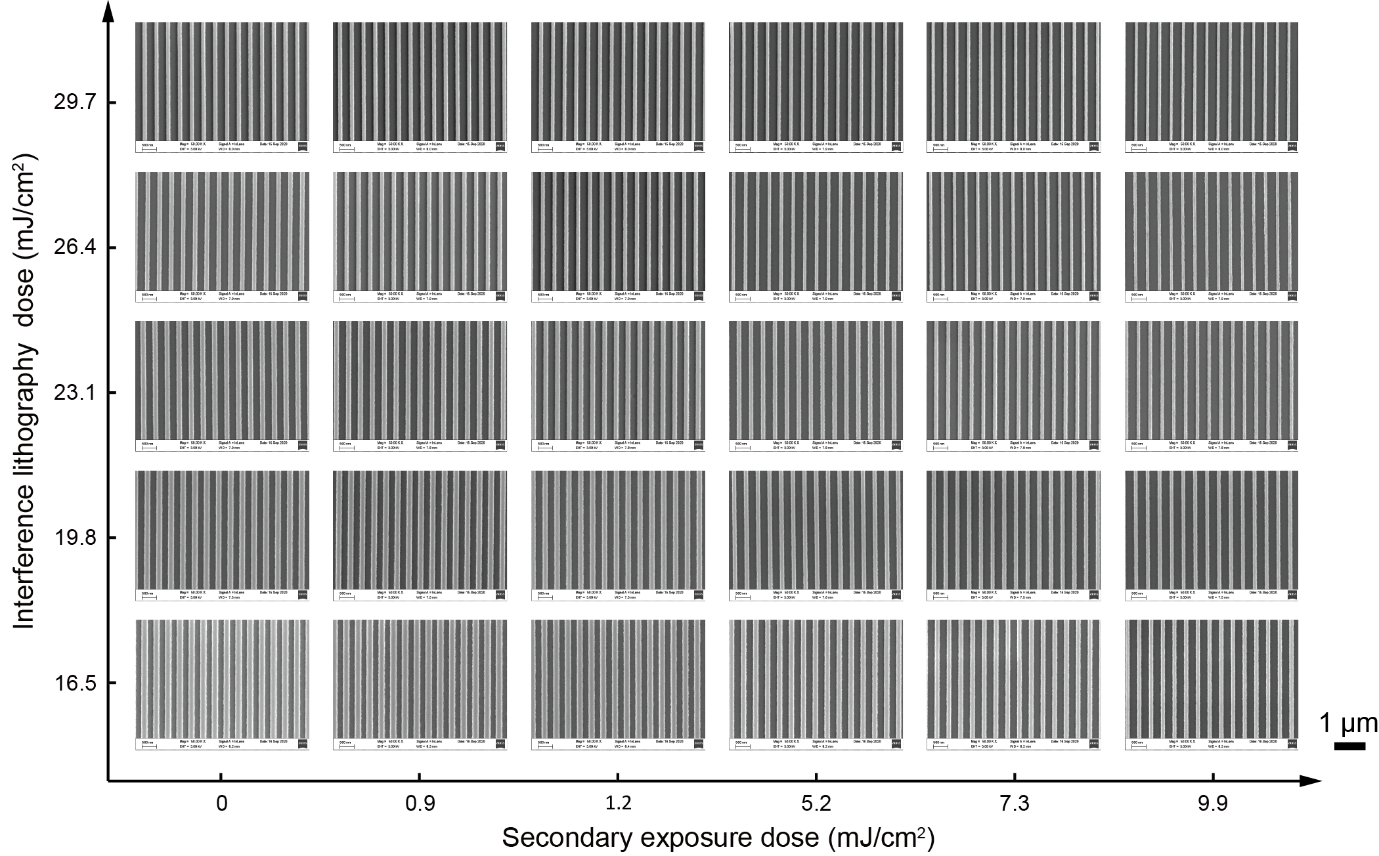
 **Fig. S10.** SEM image matrix of 400-nm-period gratings exposed by different combinations of interference lithography and secondary exposure doses.


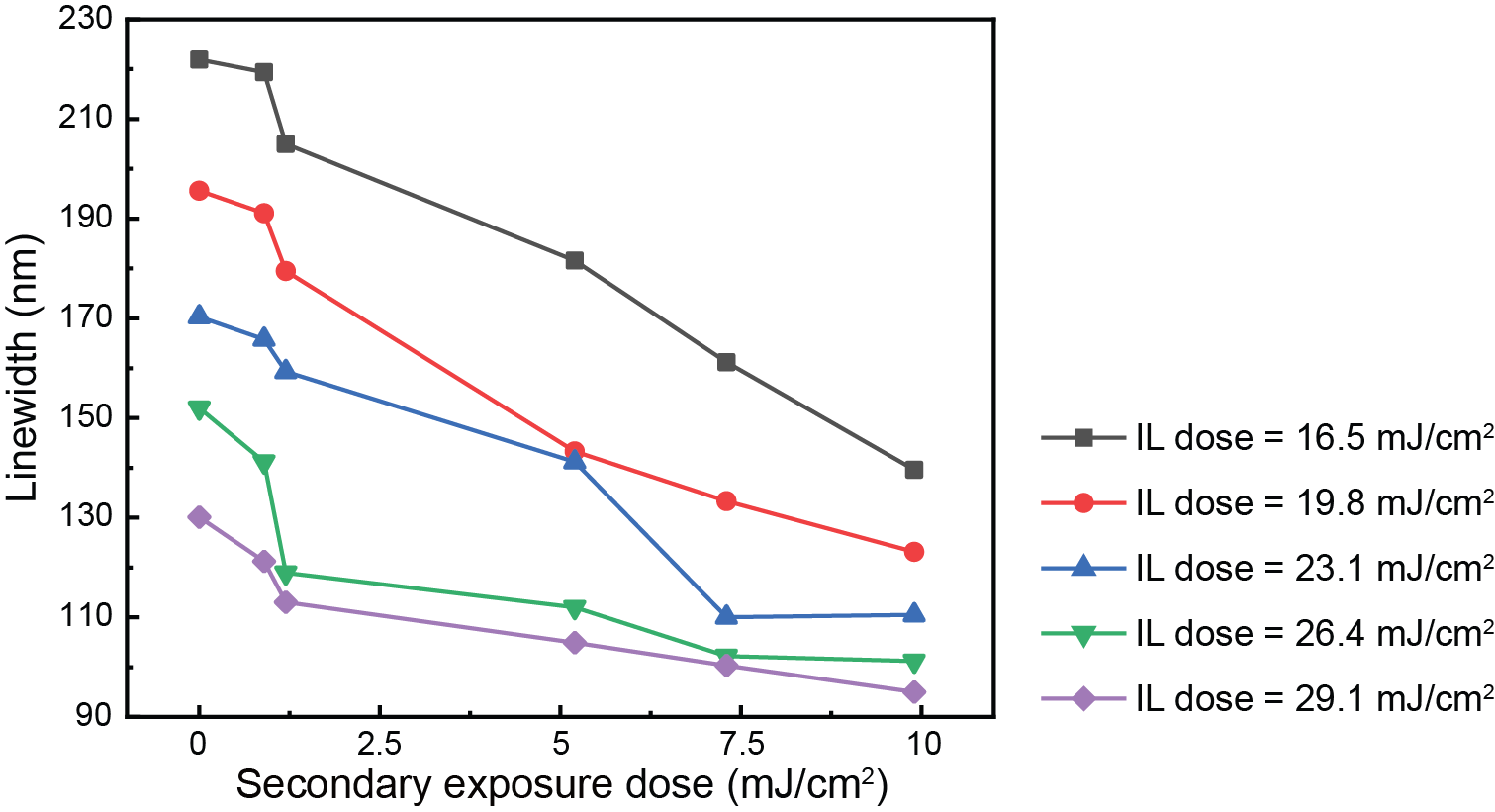


**Fig. S11.** The linewidths measured in the SEM images of **Fig. S10.**


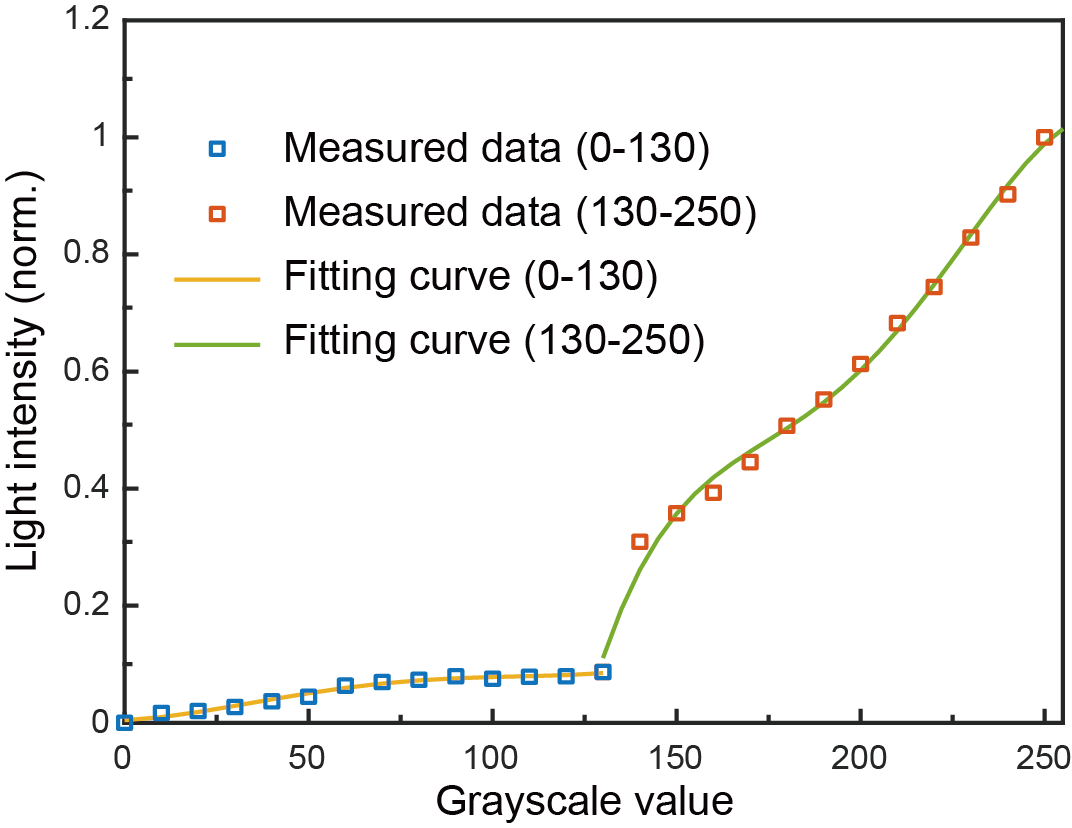


**Fig. S12.** The relationship between digital grayscale value and normalized projected light intensity using the UV projector.


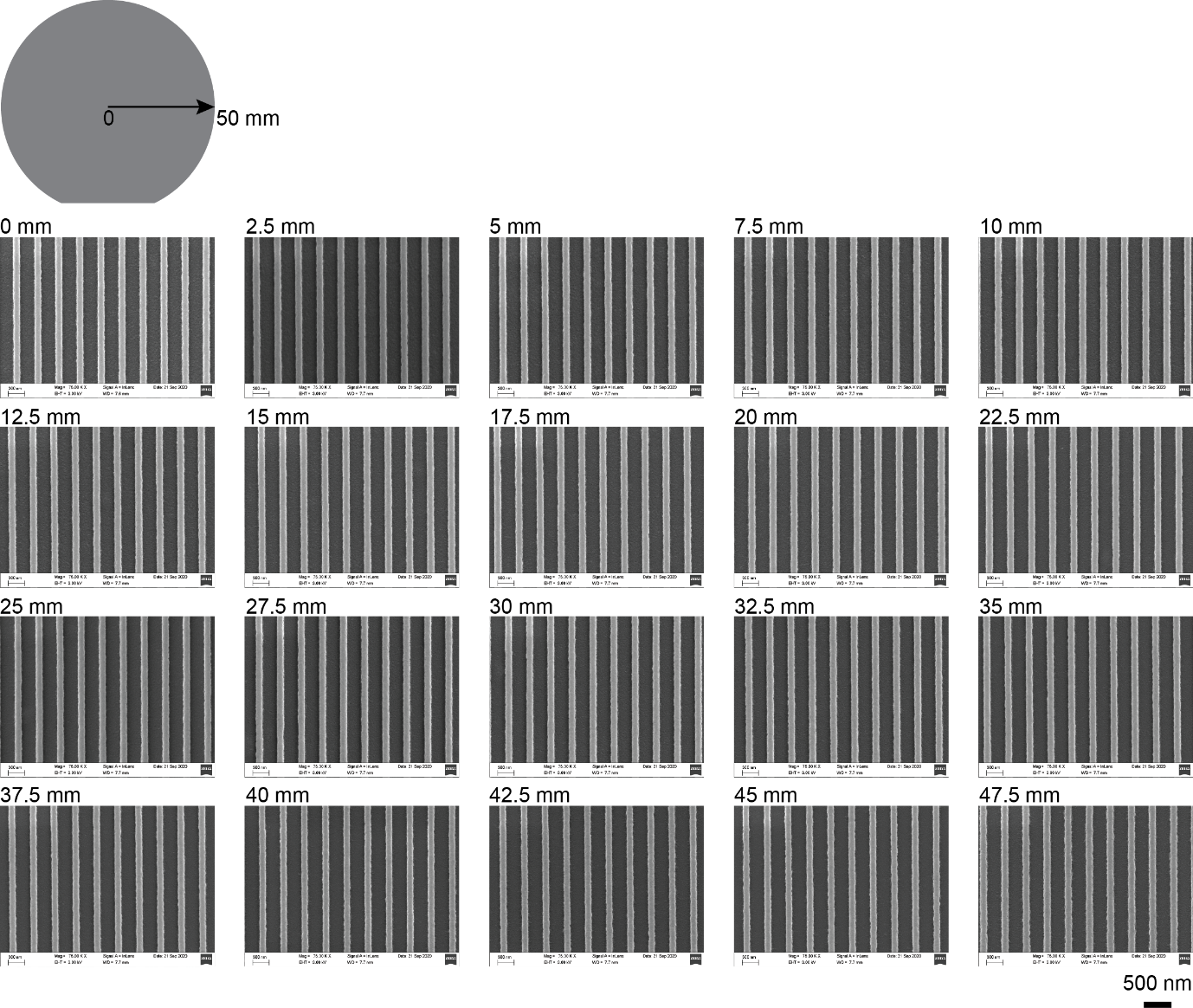


**Fig. S13.** SEM images recorded every 2.5 mm along the radius of the 4-inch wafer patterned by IL and grayscale-patterned secondary exposure.


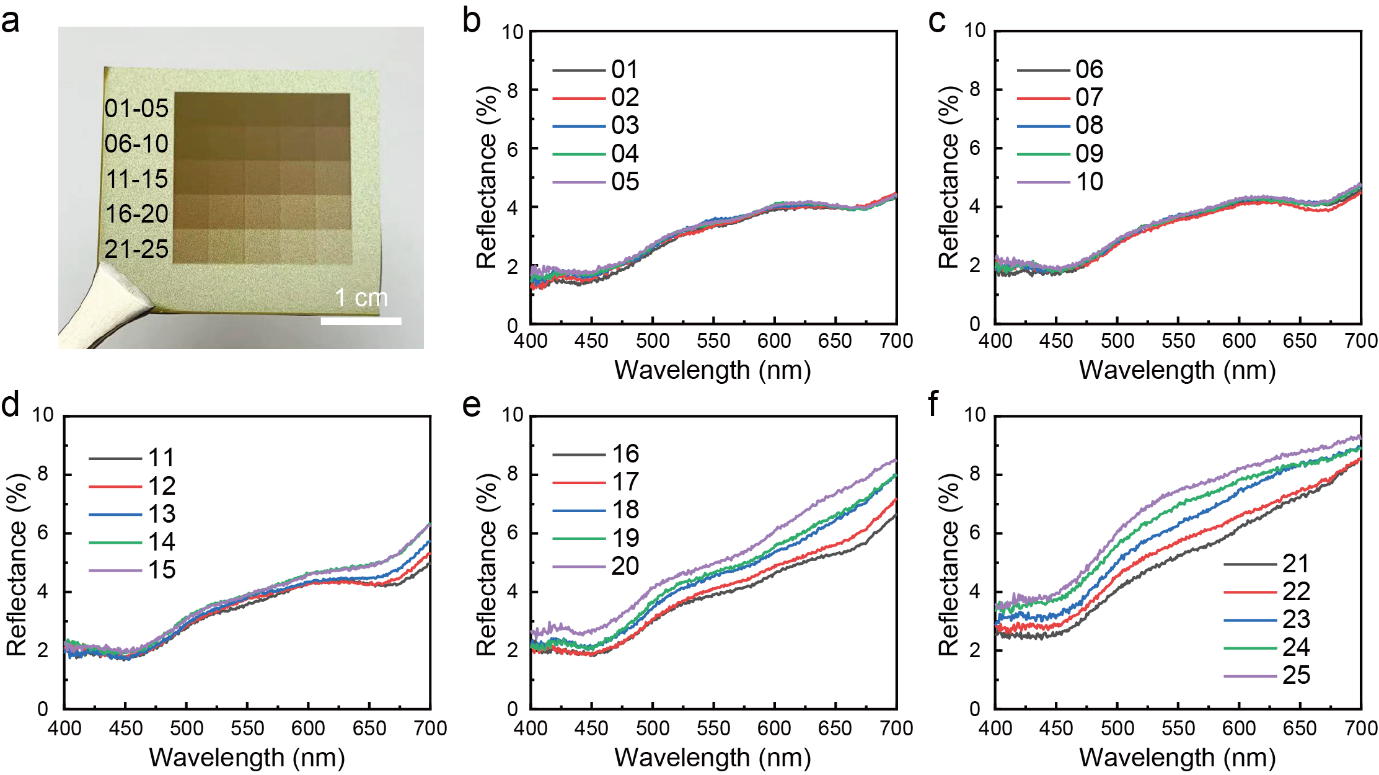


**Fig. S14.** (**a**) The photograph of 25 squares of 700-nm-period 2D patterns on photoresist modulated by secondary exposure projection, showing a clear grayscale change from dark brown to light gold. (**b-f**) Measured reflection spectra on each square.


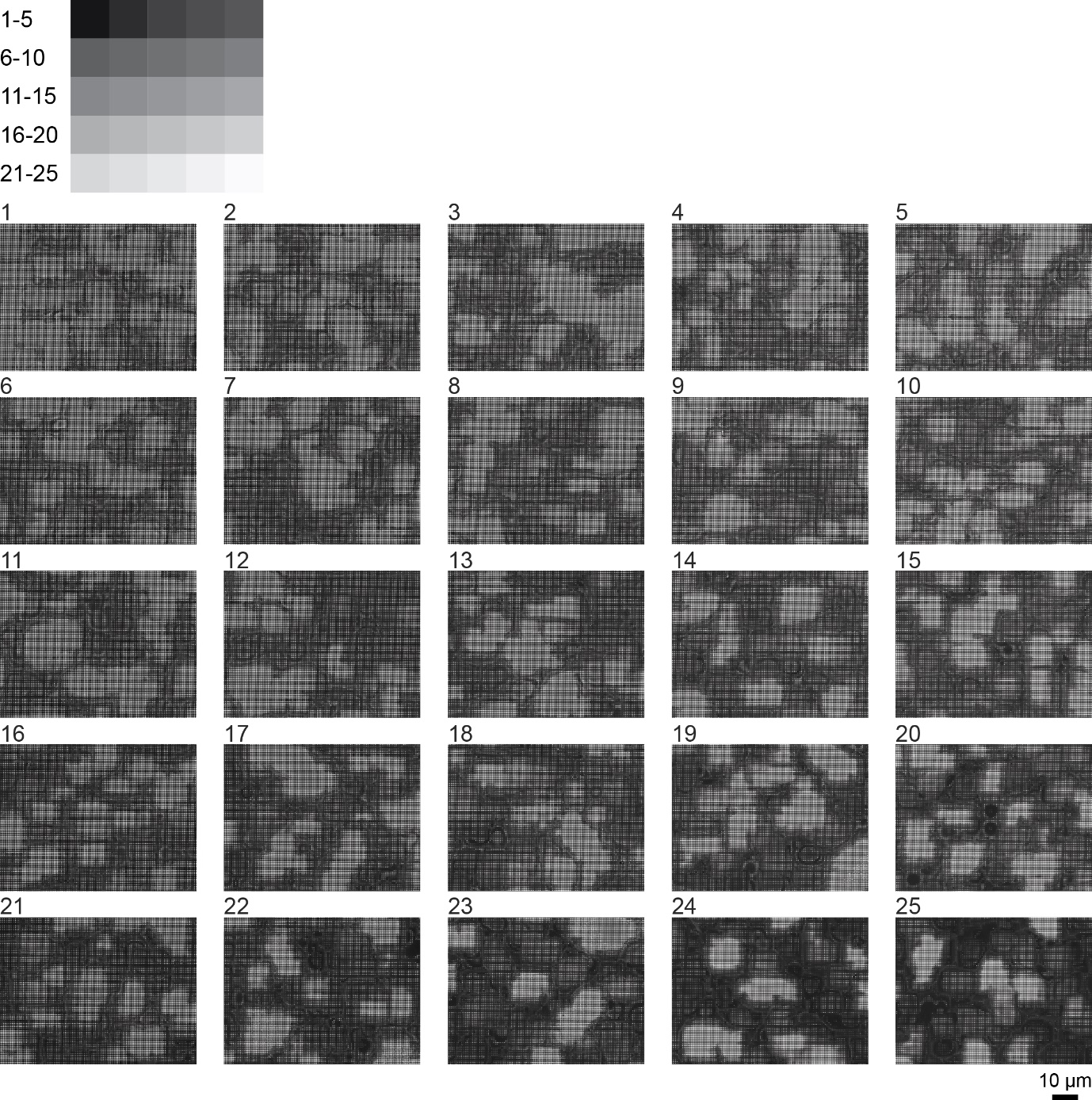
 **Fig. S15.** SEM images of all 25 areas in Figure 5b patterned by different secondary exposure doses.


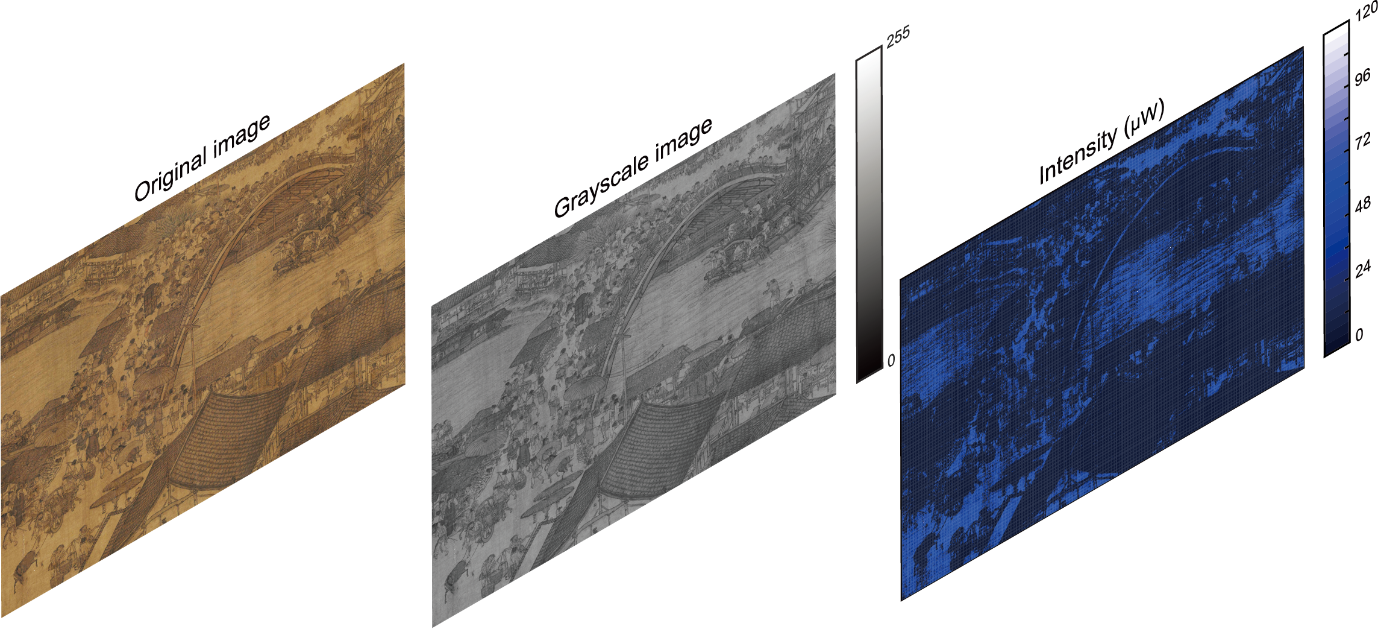


**Fig. S16.** Pixel-by-pixel mapping of an image into grayscale and intensity channels.
